# Complete Genome Sequences of Classical Swine Fever Virus Isolates Belonging to a New Subgenotype, 2.1c, from Hunan Province, China

**DOI:** 10.1128/genomeA.00080-12

**Published:** 2013-01-24

**Authors:** Da-Liang Jiang, Guo-Hua Liu, Wen-Jie Gong, Run-cheng Li, Yun-Fei Hu, Changchun Tu, Xing-Long Yu

**Affiliations:** a College of Veterinary Medicine, Hunan Agricultural University, Changsha, Hunan Province, China; b Key Laboratory of Jilin Province for Zoonosis Prevention and Control, Institute of Military Veterinary, Academy of Military Medical Sciences, Changchun, Jilin Province, China

## Abstract

Two isolates of a new classical swine fever virus (CSFV) subgenotype, 2.1c (HNLY-2011 and HNSD-2012), were recently isolated from pigs in Hunan Province, China. The most significant difference in the amino acid sequences of the polyproteins from subgenotypes 2.1a and 2.1b is an SPA → TPV amino acid substitution at positions 777 and 779 in the E2 protein.

## GENOME ANNOUNCEMENT

Classical swine fever (CSF) is a highly contagious viral disease of pigs caused by classical swine fever virus (CSFV) and is one of the Office International des Epizooties (OIE) notifiable diseases (1). CSFV is a single-stranded, positive RNA virus of the genus *Pestivirus* within the family *Flaviviridae*. Phylogenetic analysis has divided CSF viruses into three phylogenetic groups (1, 2, and 3), with each group being divided into three or four subgenotypes (1.1 to 1.3; 2.1 to 2.3; 3.1 to 3.4) (2). CSFV subgenotype 2.1 has been further divided into 2.1a and 2.1b (3, 4). In mainland China, several genotypes of CSFV strains (1.1, 2.1, 2.2 and 2.3) are largely responsible for CSF outbreaks (5), and CSFVs of subgenotype 2.1b have predominated since the 1990s (6, 7, 8). During 1989–2003 in Taiwan, a dramatic switch from subgroup 3.4 to 2.1 was observed, with subgroup 2.1a viruses predominating from 1995 onwards (4).

During 2011 and 2012, we isolated two CSFVs, HNLY-2011 and HNSD-2012, from intensive pig farms in Hunan Province, China. The full nucleotide sequences were obtained from seven overlapping fragments amplified by reverse transcription-PCR. The amplified PCR products were purified and cloned into vector PMD-18, and the recombinant plasmids were sequenced with an ABI 3730 XL genome sequencer (Sangon Company, Shanghai, China). Sequences were assembled and manually edited to produce the final genome sequences. Comparative phylogenetic analysis with 34 previous isolates (GenBank) located the two isolates into a new CSFV subgroup, 2.1c.

The complete genome sequences of isolates HNLY-2011 and HNSD-2012 are both 12,296 nucleotides (nt) in length, with a 5′ untranslated region (UTR) of 372 nt and a 3′ UTR of 227 nt. A single large open reading frame (ORF) (11,697 nt) is found in these two genomes between nt positions 373 and 12069 and is capable of coding for a polyprotein of 3,898 amino acids. The difference in the complete genome sequences between HNLY-2011 and HNSD-2012 was only 1.3%. However, a more significant level of nucleotide difference was detected as 6.4 to 7.6% and 7.1 to 7.8% between these isolates and those of subgenotypes 2.1a and 2.1b CSFVs, respectively, indicating that these two isolates are more closely related to subgenotype 2.1a than to 2.1b. Inferred amino acid sequences of the polyproteins of the two isolates, HNLY-2011 and HNSD-2012, together differed from subgenotypes 2.1a and 2.1b by 2.5 to 3.4% and 3 to 3.8%, respectively, while that between subgenotypes 2.1a and 2.1b CSFVs differed by 2.2 to 3.9%. The most significant difference in the polyproteins of HNLY-2011 and HNSD-2012 from other genotypes is an SPA → TPV amino acid substitution at positions 777 and 779 in the E2 protein. These findings provide strong evidence for substantial genetic variation within subgenotype 2.1 CSFVs, which may have implications for the prevention and control of CSFV infection in pigs. Taken together, these data support that HNLY-2011 and HNSD-2012 represent a new branch within subgenotype 2.1.

This is the first report of complete genome sequences of CSFV subgenotype 2.1c isolates. The new genome sequences may contribute to further understanding phylogeny and molecular diagnosis of CSFV.

### Nucleotide sequence accession numbers.

The complete genome sequences of CSFV HNLY-2011 and HNSD-2012 have been deposited in GenBank under accession nos. JX262391 and JX218094, respectively.
